# Understanding the Green Development Behavior and Performance of Industrial Enterprises (GDBP-IE): Scale Development and Validation

**DOI:** 10.3390/ijerph17051716

**Published:** 2020-03-05

**Authors:** Xingwei Li, Jianguo Du, Hongyu Long

**Affiliations:** 1 School of Management, Jiangsu University, Zhenjiang 212013, China; 2111710001@stmail.ujs.edu.cn; 2 School of Civil Engineering and Geomatics, Southwest Petroleum University, Chengdu 610500, China; 201821000820@stu.swpu.edu.cn

**Keywords:** green development, organizational behavior, scale development, industrial ecology, green supply chain management practice, clean production behavior, triple bottom line, factor analysis

## Abstract

Industrial enterprises have provided outstanding contributions to economic development in countries around the world. The green development of industrial enterprises has received widespread attention from researchers. However, existing research lacks the tools to scientifically measure the green development behavior and performance of industrial enterprises. According to the theory of green development behavior and performance of industrial enterprises (GDBP-IE), the aim of this paper is to provide a tool for scientifically measuring such behavior and performance. This paper determined the initial scale through literature analysis and expert discussions and obtained valid samples from 31 provincial administrative regions in China through field and online surveys (*N* = 853). The exploratory factor analysis method was used to test the reliability and validity of the scale. The main conclusions are as follows: (1) The reliability and validity of the GDBP-IE scale are good; (2) the GDBP-IE scale, with a total of 70 items, comprises four sub-scales: The internal factors sub-scale, the external factors sub-scale, the green development behavior of industrial enterprises sub-scale, and the green development performance of industrial enterprises sub-scale. Among them, the internal factors sub-scale, with a total of 13 items, consists of two dimensions: Corporate tangible resources and corporate intangible resources. The external factors sub-scale, with a total of 23 items, consists of three dimensions: Market environment; public supervision; policy and institutional environment. The green development behavior of industrial enterprises sub-scale, with a total of 18 items, consists of two dimensions: Clean production behavior and green supply chain management practice. The green development performance of industrial enterprises sub-scale, with a total of 16 items, comprises three dimensions: Corporate social performance, corporate financial performance, and corporate environmental performance. The findings enrich the research on corporate organizational behavior, green behavior, and green development system theory, and provide tools for further empirical testing. The development and verification of green development behavior and performance of industrial enterprises can help guide various types of industrial enterprises in transforming to green development and can provide a reference for the government to formulate targeted green development policies.

## 1. Introduction

Since the publication of *Our Common Future* in 1987, the importance of sustainable development has been recognized by researchers around the world [1]. Sustainable development is defined as development that meets the need of the present generation without compromising the needs of future generations. To guide the global efforts of sustainable development in 2015–2030, the United Nations Development Programme breaks down sustainable development goals into 17 detailed goals: No poverty; zero hunger; good health and well-being; quality education; gender equality; clean water and sanitation; affordable and clean energy; decent work and economic growth; industry, innovation, and infrastructure; reduced inequalities; sustainable cities and communities; responsible consumption and production; climate action; life below water; life on land; peace, justice, and strong institutions; and partnerships for the goals [2].

In recent years, research on green development (GD) has attracted researchers’ attentions. Green development is a complex adaptive system closely related to the social, economic, and natural environment. It is affected by production, life, policies, and the GD concept, and its core is the human and natural life communities [3,4,5]. Whereas both GD and sustainable development focus on the natural environment and economic development, GD emphasizes the systematism of the economy and natural environment.

According to data from the National Bureau of Statistics of China [6], from 2016 to 2018, China’s industrial added value accounted for more than 40% of GDP. Industrial enterprises have provided outstanding contributions to the economic development of many countries, including China. However, when promoting economic development, the organizational behavior of industrial enterprises can easily produce negative environmental impacts. This characteristic is particularly prominent in developing countries, for example, China [3], India [7], and Malaysia [8]. According to data surveyed by the National Bureau of Statistics of China [9], annual industrial solid waste generation in China is 3159.92 million tons, of which 54.64% is comprehensively used. In other words, Chinese industrial enterprises still need to accelerate the transition to green development and reduce their negative impact on the environment [10]. To reduce the negative environmental impact of economic development, GD policies have emerged at a historic moment and have received responses from countries around the world, especially China [11].

Many researchers have evaluated various aspects of the performance of GD, for example, green industry performance index and green industry progress index [12], green total-factor growth performance [13], green total-factor productivity [14], efficiency of innovation and environmental performance [15], green innovation performance [16], organizational performance and environmental performance of green human resource management [17], and green supply chain management practices and performance [18,19]. Although these studies deeply enriched the field literature on GD performance, a scientific and effective tool is lacking to measure the GD behavior and performance of industrial enterprises (GDBP-IE).

For a long time, the relationship between corporate organizational behavior and performance was the focus of many management researchers [20,21,22]. The theory of GDBP-IE states that in order to respond to environmental protection and enterprise development, the green behavior formed by industrial enterprises as an organizational carrier is called the GD behavior of industrial enterprises, including clean production behavior and green supply chain management practices [23]. Thus, how is it possible to scientifically and effectively measure and verify GDBP-IE? To answer the question, we aimed to provide a scientific tool for measuring GDBP-IE according to the GDBP-IE theory.

Scale development can be used to scientifically and effectively measure emerging academic concepts and has been widely recognized in the fields of environmental psychology and environmental management [24,25,26,27,28,29,30,31]. Therefore, according to the procedures of scale development, we created a GDBP-IE scale with four sub-scales using a questionnaire survey and an exploratory factor analysis method, and tested its reliability and validity. To the best of our knowledge, this is the first time that a GDBP-IE scale has been developed and verified. 

The remainder of this paper is organized as follows. Section 2 reviews relevant literature in terms of three aspects: Influencing factors, the green development behavior of industrial enterprises (GDB-IE), and the green development performance of industrial enterprises (GDP-IE). Section 3 describes the steps involved in scale development, the exploratory factor analysis method, and data sources. Section 4 describes the results of the exploratory factor analysis, including the principal component analysis, validity analysis, and reliability analysis. Section 5 introduces the similarities and differences between similar research results and this study, and objectively outlines the limitations and future research directions. The final section summarizes the main new findings and management implications.

## 2. Literature Review

Industrial enterprises are affected by production, life, and policies. Therefore, the GD of industrial enterprises has been increasingly valued by researchers [12,13,14,15,16,17,18,19]. This section provides a review of the relevant literature in terms of three aspects: Influencing factors, GDB-IE, and GBP-IE. Table 1 shows the relevant literature on the GD behavior and performance of industrial enterprises.

### 2.1. Internal Factors (IFs)

The IFs of an enterprise are composed of enterprise resources. Resource-based view of the firm was first proposed by Wernerfelt [32], and then continuously developed and improved by researchers [33]. As a competitive advantage of an enterprise, resources can be used to distinguish the level of enterprise performance [34]. According to a resource-based view of the firm, the enterprise is regarded as a collection of resources, and a powerful framework was proposed to unify and combine several different resources to generate a competitive advantage. However, no uniform standard exists for the definition or classification of resources in academia [35]. For example, some studies suggested that enterprise resources include tangible, intangible, and human resources [36], whereas others suggested that enterprise resources include only tangible and intangible resources [10,23,37]. The key to these research disputes is human resources. From a macro perspective, however, tangible resources can explain the scope of human resources. Per previous research, dividing resources into tangible and intangible resources is a generally accepted paradigm.

Corporate tangible resources refer to visible and tangible resources having a physical form, such as machinery, equipment, workshops, and raw materials owned by the enterprise [38,39]. In a resource-based view of the firm, tangible resources are an important factor in the study of corporate performance because of their controllability. Newbert [40] stated that tangible resources must be considered in the actual research of the resource-based theory of the firm, which can not only directly result in high performance output but also produce a competitive advantage [41].

Corporate intangible resources are resources that do not have a physical form but can provide certain rights to the enterprise, such as patent rights, land use rights, non-patented technologies, copyrights, trademark rights, and reputation [42,43]. Corporate intangible resources are vital to the survival and development of an enterprise and directly impact the competitiveness of the enterprise [38]. Newbert [40] thought that intangible resources play the greatest role in the success of enterprises. Therefore, the phenomenon of "weak advantage over strong" in the market can be understood. The competence-based theory of the firm showed that corporate capabilities played an important role in corporate performance [44]. According to a resource-based view of the firm, capabilities are an important part of corporate intangible resources. However, intangible resources are dual in nature, and their existence can also place enterprises at a competitive disadvantage or even result in bankruptcy [45].

At present, corporate tangible and intangible resources are considered to be the internal factors of the enterprise. However, the existing studies lack a comprehensive consideration of the IFs scale regarding GDB-IE. In order to overcome for this shortcoming, based on previous studies, we comprehensively considered GDB-IE and developed an IFs sub-scale.

### 2.2. External Factors (EFs)

Institutions are artificially designed restrictions that constitute political, economic, and social communication, including informal institutions (e.g., normative constraints, taboos, customs, traditions, and codes of conduct) and formal institutions (e.g., the constitution, laws, and property rights) [46]. The instability and unsustainability of the institutions can increase transaction costs, whereas the improvement of the institution environment can reduce transaction costs and improve transaction efficiency [47]. Enterprise production and operation activities are embedded in a certain institutional environment. Therefore, the policy and institutional environment is closely related with the development strategy of the enterprise.

In their theory of Industrial Organization, Bain [48,49] advocated researching industrial economic issues with the structure–conduct–performance paradigm. The analysis framework dictates that a certain market structure affects market behavior and then market performance. In this regard, Stigler et al. [50] hold different views. They think that an adverse impact relationship may exist among the three-fold paradigm, that is, changes in market performance will adversely affect market behavior and market structure. If the transformation of GD achievements of industrial enterprises is regarded as a kind of market behavior, the influence of a certain market structure and market performance on market behaviors can be analyzed (i.e., transformation of GD achievements of industrial enterprises) [51]. Therefore, the market environment is important in the GD of industrial enterprises.

From historical and intellectual perspectives, in developing countries like China, to promote the GD of industrial enterprises, public supervision is indispensable. Public supervision can compensate for the deficiencies of the legal system and play a role in transmitting information to enterprises, thereby promoting enterprises to GD [52]. Generally, the public can obtain relevant information through television, radio, newspapers, the Internet, and other media, and indirectly exert pressure on enterprises through public opinion [53]. Wu [54] found that as a third-party independent supervisor, the news media are an important driving force for enterprises to actively fulfill their social responsibilities. Porter et al. [55] considered the media and government agencies as important sources of corporate responsibility for the consequences of their actions. In addition, to maintain the enterprise’s good reputation and social image, the management of industrial enterprises will actively undertake social responsibilities through GD and positively respond to relevant media reports [56,57].

However, the existing studies lack an EFs scale that comprehensively considers GDB-IE. In the context of China, tools for measuring policy and institutional environments, market environments, and public supervision through scales have not yet been developed. To address this shortcoming, we comprehensively considered GDB-IE and developed an EFs sub-scale based on previous studies.

### 2.3. Green Development Behavior of Industrial Enterprises (GDB-IE)

The green development behavior of industrial enterprises has attracted increasing attention from environmental management researchers. The green production behavior of industrial enterprises plays an important role in production, management, and consumption [58,59]. Li et al. [23] constructed a theoretical model of GDB-IE through research on grounded theory of Chinese industrial enterprises. In this study, we considered GDB-IE is embodied in clean production behavior [60,61] and green supply chain management practices [62,63]. Although clean production behavior cannot produce economic benefits in the short term, long-term clean production technology innovation can change the production costs of enterprises and enhance corporate competitive advantages. To improve environmental performance, enterprises are subject to government regulation in the short term and passively increase investment in environmental technology upgrades. However, forcing enterprises to increase costs can damage competitive advantages [64,65].

With the continuous deterioration of the resources and environment, the contradiction amongst economic development, environmental protection, and social development has become increasingly prominent. Enterprise operation management models need to seek a path to GD. Therefore, green supply chain management has become an important operational strategy for the GD of enterprises [66,67]. Since the 20th century, some leading enterprises have implemented green supply chain management and achieved significant environmental and economic benefits. Green supply chain management practices are considered to be traditional supply chain activities that limit the harmful effects on services or production products throughout the life cycle, including green manufacturing, green packaging, and reverse logistics [68,69,70,71].

However, existing studies lack a scale for GDB-IE. In particular, in the Chinese context, tools for measuring clean production behavior and green supply chain management practice through scales have not yet been developed. To address this shortcoming, we developed a GDB-IE sub-scale for GDB-IE based on previous studies.

### 2.4. Green Development Performance of Industrial Enterprises (GDP-IE)

The triple bottom line theory considers corporate financial performance, corporate environmental performance, and corporate social performance in a framework [72], and the theory is accepted by researchers in the field of GD [23,73,74,75,76].

Although most researchers support studies on enterprise performance from the perspective of supply chain management practices, a comprehensive consideration of GDB-IE and its influencing factors is lacking. Heterogeneity exists between different industries, and the GD opportunities and challenges faced by different industries are different. The GD of industrial enterprises is not completely consistent with that of other enterprises. Therefore, the introduction of triple bottom line theory into the research of industrial enterprises’ GD behavior and performance and the influencing factors can compensate for this deficiency. In addition, researchers engaged in GD performance research mostly focus on the construction and evaluation of indicator systems but have not provided a measurable scale tool. To address this limitation, we comprehensively considered GDB-IE and its influencing factors based on previous studies, and developed a GDP-IE sub-scale.

## 3. Materials and Methods

First, the main structure of the scale was determined through literature analysis. According to the theoretical model of industrial enterprises’ GD behavior and performance constructed by Li et al., we determined that the scale consists of four parts: Internal factors, external factors, GD behavior of industrial enterprises, and GD performance of industrial enterprises [23]. The items of internal factors sub-scale were sourced from previous studies [77,78,79,80,81], the external factors sub-scale items were mainly sourced from [51,82], those of GD behavior of industrial enterprises sub-scale were mainly obtained from [79,80,83], and for the items of GD performance of industrial enterprises sub-scale, we mainly referred to references [77,84,85].

**Figure 1:**
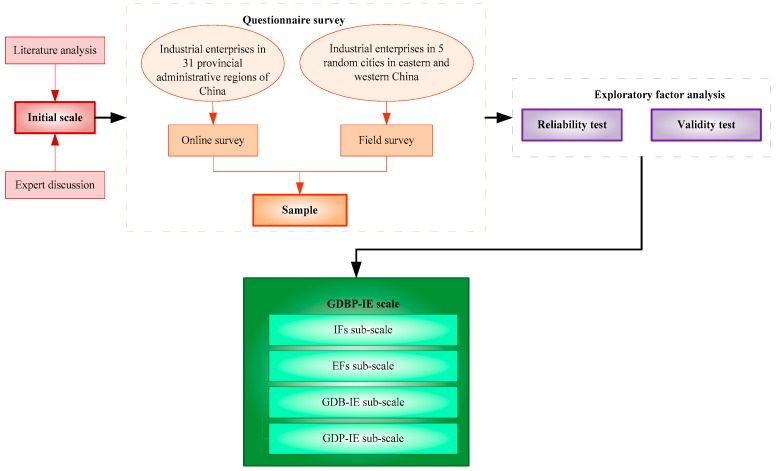
Framework for GDBP-IE scale development. Note: IFs—internal factors; EFs—external factors; GDB-IE—green development behavior of industrial enterprises; GDP-IE—green development performance of industrial enterprises.

Second, in July 2018, initial item pools were evaluated and affirmed through a meeting discussion. The researchers involved in the discussion consisted of 3 professors and 2 doctors and met the following two conditions: (1) From the field of management science; (2) familiar with the scale development process. Anonymous voting was used to vote for all items from the literature and self-developed items, and items with less than four votes were deleted. Finally, a five-level Likert scale containing 70 items was obtained as the initial scale: IFs sub-scale (13 items), EFs sub-scale (23 items), GDB-IE sub-scale (18 items), and GDP-IE sub-scale (16 items). Figure 1 shows the research framework.

### 3.1. Data Sources

Using a random sampling strategy, two methods were used to obtain samples: Field survey and online survey. From September 2018 to June 2019, we randomly selected workers and managers of industrial enterprises from five cities in Eastern and Western China (i.e., Zhenjiang, Wuxi and Changzhou in Jiangsu Province, and Chengdu and Luzhou in Sichuan Province) and issued 600 questionnaires. According to survey data from the National Bureau of Statistics of China [1], the industrial sales value of industrial enterprises above the designated size in Jiangsu and Sichuan in 2016 were the highest values in Eastern and Western China. Therefore, we chose Jiangsu and Sichuan provinces for our field research. During this period, 400 questionnaires were issued to industrial workers and managers from 31 provinces in China (excluding Hong Kong, Macau, and Taiwan) through online surveys using a commissioned questionnaire website www.wjx.cn. After removing the unqualified questionnaires, 853 valid samples were finally obtained, and the effective rate was 85.3%. Table 2 provides descriptive statistics of the samples.

The results are shown in Table 2. (1) From the perspective of sex distribution, 572 male (67.06%) and 281 female (32.94%) employees were included in the sample. The proportion of men was slightly higher than that of women, which is consistent with the sex distribution characteristics of industrial enterprises in actual work. (2) From the perspective of age distribution, people aged under 30, 30–39, 40–49 years, and older than 50 years were 250 (29.31%), 366 (42.91%), 177 (20.75%), and 60 (7.03%), respectively. The samples were mainly middle-aged and young people, which is consistent with the age distribution characteristics of industrial enterprises in actual work. (3) From the perspective of position distribution, workers and managers in the sample accounted for 364 (42.67%) and 489 (57.33%), respectively. The difference between the proportion of workers and managers in the sample is small, but the proportion of managers is slightly higher than workers. In the organization, managers have the right to make management decisions on organizational behavior, which is in line with the characteristics of management decisions of industrial enterprises in actual work. (4) From the perspective of level of education, 494 (57.91%) employees had bachelor’s degrees in the sample and 359 (42.09%) had other degrees. The samples were dominated by bachelor’s degrees, which is consistent with the level of education characteristics of industrial enterprises in the actual workplace. (5) From the perspective of the number of employees in an enterprise, those working in three types of enterprises (i.e., less than 300, 301–1000, and more than 1000 employees) were 309 (36.23%), 310 (36.34%), and 234 (27.43%), respectively. In other words, the samples covered large, medium, and small industrial enterprises. Therefore, the sample was representative.

### 3.2. Exploratory Factor Analysis

The exploratory factor analysis method was used to test the reliability and validity of the scale. Exploratory factor analysis is a common method used for scale development, including reliability tests and validity tests. We used SPSS 25.0 software (International Business Machines Corporation, Armonk, NY, USA) to test the reliability and validity of the scale. In the analysis process, the following criteria were used to determine whether the items were reasonable:

(1) Kaiser–Meyer–Olkin (KMO) value. Kaiser [86] stated that, “when the KMO value is in the 0.90s, marvelous; in the 0.80s, meritorious; in the 0.70s, middling; in the 0.60s, mediocre; in the 0.50s, miserable; below 0.50, unacceptable”. In other words, if a factor analysis is suitable, the KMO value of the questionnaire must be greater than 0.6.

(2) Factor loadings. Factor loadings reflect the importance of the item to the extracted common factor, and the value cannot be less than 0.4.

(3) Communality. Communality is the proportion of variation the item can explain for a common trait or attribute.

(4) Cronbach’s α coefficient value. The Cronbach’s α coefficient value is an index used to judge the internal consistency of the questionnaire in reliability analysis. When the Cronbach’s α coefficient value is above 0.9, the reliability of the scale is ideal; when between 0.8 and 0.899, the reliability of the scale is very good; when between 0.7 and 0.799, the reliability of the scale is good; when between 0.6 and 0.699, the reliability of the scale is acceptable; when between 0.5 to 0.599, the reliability of the scale is acceptable but low; when less than 0.5, the reliability of the scale is unacceptable.

(5) Corrected item and total correlation. The corrected item and total correlation coefficient value is an index used for judging the internal consistency of the item and the remaining items. If the coefficient value is less than 0.4, the internal consistency of the item and the remaining items is low.

## 4. Results

Validity was tested by KMO and Bartlett’s test of sphericity. Table 3 shows the KMO and Bartlett’s test of sphericity of the scale.

The results in Table 3 show that the KMO values of the IFs sub-scale, EFs sub-scale, GDB-IE sub-scale, and GDP-IE sub-scale were 0.938, 0.930, 0.948, and 0.935, respectively, all of which are higher than 0.9. In addition, the significance of Bartlett’s test of sphericity for the scale was less than 0.001. Therefore, common factors exist among the variables, which indicates suitability for further exploratory factor analysis. Next, we analyzed the principal components and reliability of the scale separately.

### 4.1. Development and Verification of IFs sub-Scale

#### 4.1.1. Principal Component Analysis (PCA)

Table 4 shows the principal component and factor loadings of the IFs sub-scale. The factor loadings were between 0.559 and 0.799 (higher than 0.4); the communality was between 0.488 and 0.681 (higher than 0.2), indicating that the scale is acceptable with a good interpretation reliability. Therefore, two factors of the IFs sub-scale were obtained through PCA, that is, corporate tangible resources (including nine items) and corporate intangible resources (including four items).

#### 4.1.2. Reliability Analysis

Table 5 shows the reliability test results of the IFs sub-scale. The Cronbach’s α coefficient of the IF sub-scale was 0.908 (above 0.9), indicating that the overall internal consistency of the scale is ideal. In addition, the Cronbach’s α coefficients of the two factors with corporate tangible resources and corporate intangible resources were 0.888 and 0.808 (above 0.8), respectively, indicating that the internal consistency of the factors is very good.

### 4.2. Development and Verification of EFs Sub-Scale

#### 4.2.1. PCA

Table 6 shows the principal component and factor loadings of the EFs sub-scale. The factor loadings were between 0.466 and 0.759 (higher than 0.4); the communality was between 0.412 and 0.659 (higher than 0.2), which indicates that the scale is acceptable with a good interpretation reliability. Therefore, three factors of the EFs sub-scale were obtained through principal component analysis, that is, market environment (including 11 items), public supervision (including eight items), and policy and institutional environment (including four items).

#### 4.2.2. Reliability Analysis

Table 7 shows the reliability test results of the EFs sub-scale. The Cronbach’s α coefficient of the EFs sub-scale was 0.920 (higher than 0.9), indicating that the overall internal consistency of the scale is ideal. In addition, the Cronbach’s α coefficients of the three factors market environment, public supervision, and policy and institutional environment were 0.869, 0.845, and 0.827 (higher than 0.8), respectively, indicating that the internal consistency of the factors is very good.

### 4.3. Development and Verification of GDB-IE Sub-Scale

#### 4.3.1. PCA

Table 8 shows the principal component and factor loadings of the GDB-IE sub-scale. The factor loadings were between 0.409 and 0.812 (higher than 0.4); and communality was between 0.266 and 0.672 (higher than 0.2), indicating that the scale is acceptable with a good interpretation reliability. Therefore, two factors of the GDB-IE sub-scale were obtained through principal component analysis: Clean production behavior (including 11 items) and green supply chain management practices (including eight items).

#### 4.3.2. Reliability Analysis

Table 9 shows the reliability test results of the GDB-IE sub-scale. The Cronbach’s α coefficient of the GDB-IE sub-scale was 0.925 (above 0.9), indicating that the overall internal consistency of the scale is ideal. In addition, the Cronbach’s α coefficients of the two factors clean production behavior and green supply chain management practices were 0.880 and 0.866 (above 0.8), respectively, indicating that the internal consistency of each factor is very good.

### 4.4. Development and Verification of GDP-IE Sub-Scale

#### 4.4.1. PCA

Table 10 shows the principal component and factor loadings of the GDP-IE sub-scale. The factor loadings were between 0.543 and 0.824 (higher than 0.4); and communality was between 0.450 and 0.752 (higher than 0.2), indicating that the scale is acceptable with a good interpretation reliability. Therefore, three factors of the GDP-IE sub-scale were obtained through principal component analysis: Corporate social performance (including six items), corporate financial performance (including six items), and corporate environmental performance (including six items).

#### 4.4.2. Reliability Analysis

Table 11 shows the reliability test results of the GDP-IE sub-scale. The Cronbach’s α coefficient of the GDP-IE sub-scale was 0.909 (above 0.9), indicating that the overall internal consistency of the scale is ideal. In addition, the Cronbach’s α coefficients of corporate social performance, corporate financial performance, and corporate environmental performance were 0.845, 0.833, and 0.834 (above 0.8), respectively, indicating that the internal consistency of the factors is very good.

## 5. Discussion

In the fields of environmental psychology and environmental management, exploratory factor analysis can be used to effectively measure the reliability and validity of factors. Through exploratory factor analysis, we found that the reliability and validity of the GDBP-IE scale is very good. To the best of our knowledge, this is the first time that a GDBP-IE scale has been developed and verified.

We found that the GDBP-IE scale consists of four parts: IF, EF, GDB-IE, and GDP-IE. This is consistent with the conclusions of Li et al. [23]. However, Li et al. [23] only proposed a theoretical model without providing a method for measuring GDBP-IE. The GDBP-IE scale developed here effectively compensates for this deficiency and provides a scientific basis for further research on the mechanism of the GDBP-IE scale.

The GDBP-IE scale comprises IF, EF, GDB-IE, and GDP-IE sub-scales. The IFs sub-scale is composed of two dimensions: Corporate tangible resources and corporate intangible resources. This is consistent with the research by Newbert [40] and Muller and Kolk [41]. The EFs sub-scale has three dimensions: Market environment, public supervision, and policy and institutional environment. This is consistent with the research by North [46], Jaiswal and Kant [51], and Porter et al. [55]. The GDB-IE sub-scale is composed of two dimensions: Clean production behavior and green supply chain management practices. This is consistent with the research by Qin et al. [64] and Ahi and Searcy [67]. The GDP-IE sub-scale has three dimensions: Corporate social performance, corporate financial performance, and corporate environmental performance. This is consistent with the research by Elkington [73], Ahmed and Sarkar [76], and Li et al. [23].

This study inevitably has some limitations. The samples in this paper are from Chinese industrial enterprises only, so may not be applicable to other areas. Therefore, on the basis of our findings, the sample scope can be expanded to include other countries and regions in the future. Although we developed and validates the GDBP-IE scale, we did not consider agriculture or services. Therefore, on the basis of our scale, new scales can be developed in the future based on the characteristics of the agriculture or service industries.

## 6. Conclusions and Implications

### 6.1. Conclusions

According to the theory of GDBP-IE, we developed and validated the GDBP-IE scale (Appendix A, Table A1). First, the initial scale was determined through literature analysis and expert discussion. Then, through field surveys and online surveys, valid samples were obtained from 31 provincial administrative regions in China (*N* = 853). Finally, the exploratory factor analysis method was used to test the reliability and validity of the scale. The main conclusions are as follows:

(1) The reliability and validity of the GDBP-IE scale is very good.

(2) The GDBP-IE scale consists of four sub-scales with a total of 70 items: IFs, EFs, GDB-IE, and GDP-IE sub-scales. Among them, the IFs sub-scale is composed of two dimensions with a total of 13 items: Corporate tangible resources and corporate intangible resources. The EFs sub-scale comprises three dimensions with a total of 23 items: Market environment, public supervision, and the policy and institutional environment. The GDB-IE sub-scale is composed of two dimensions—clean production behavior and green supply chain management practices—with a total of 18 items. The GDP-IE sub-scale comprises three dimensions—corporate social performance, corporate financial performance, and corporate environmental performance—with a total of 16 items. 

### 6.2. Implications

The theoretical and practical implications of our findings are as follows: 

(1) Theoretically, as an emerging field in corporate organizational behavior, the development and verification of the GDBP-IE scale helps enrich the literature on corporate organizational behavior. As an emerging field in green behavior, the development and verification of the GDBP-IE scale can help enrich green behavior theory. In addition, the development of the GDBP-IE scale can help further reveal the mechanism of the production subsystem in the GD system [3,87] and provide a tool for further empirical inspection. 

(2) Practically, the development and verification of the GDBP-IE scale can help to correctly guide various types of industrial enterprises to transform to GD and provide a reference for the government to formulate targeted GD policies.

## Figures and Tables

**Table 1:** Relevant literature on the green development (GD) behavior and performance of industrial enterprises.

Fields	Theoretical Basis	Main Points	References
Internal factors (IFs)	Resource-based view of the firm [32,33,34,35,36,37]; resource-based theory of the firm [38,39,40,41]; competence-based theory of the firm [42,43,44,45]	Corporate tangible resources	[38,39,40,41]
		Corporate intangible resources	[42,43,44,45]
External factors (EFs)	Theory of industrial organization [46,47,48,49,50,51,52,53,54,55,56,57]	Policy and institutional environment	[46,47]
		Market environment	[48,49,50,51]
		Public supervision	[52,53,54,55,56,57]
Green development behavior of industrial enterprises (GDB-IE)	Theory of green development behavior and performance of industrial enterprises (GDBP-IE) [23,58,59,60,61,62,63,64,65,66,67,68,69,70,71]	Clean production behavior	[23,58,59,60,61,62,63,64,65]
		Green supply chain management practice	[23,66,67,68,69,70,71]
Green development performance of industrial enterprises (GDP-IE)	Triple bottom line [72,73,74,75,76]	Corporate financial performance	[23,72,73,74,75,76]
		Corporate environmental performance	[23,72,73,74,75,76]
		Corporate social performance	[23,72,73,74,75,76]

**Table 2:** Descriptive statistics (*N* = 853).

Socio-Demographic Factors	No. of Participants	Proportion
Sex		
Male	572	67.06%
Female	281	32.94%
Age		
<30	250	29.31%
30–39	366	42.91%
40–49	177	20.75%
>50	60	7.03%
Position		
Worker	364	42.67%
Manager	489	57.33%
Level of education		
Bachelor’s degrees	494	57.91%
Other	359	42.09%
Number of employees in the enterprise		
<300	309	36.23%
301–1000	310	36.34%
>1000	234	27.43%

**Table 3:** Kaiser–Meyer–Olkin (KMO) and Bartlett’s test of sphericity.

Index		IFs Sub-Scale	EFs Sub-Scale	GDB-IE Sub-Scale	GDP-IE Sub-Scale
KMO measure of sampling adequacy		0.938	0.930	0.948	0.935
Bartlett’s test of sphericity	Approx. Chi-Square	4988.514	8120.811	7093.649	6217.588
	df	78	253	153	120
	Sig.	0.000	0.000	0.000	0.000

Note: df—degrees of freedom; Sig.—significance.

**Table 4:** Principal component and factor loadings of IFs sub-scale (*N* = 853).

Item	Component		Communality
	Corporate Tangible Resources	Corporate Intangible Resources	
Our enterprise is an organization with a high awareness and mission of green production.	0.779		0.632
Our enterprise has a leadership that values green production.	0.740		0.600
Our enterprise has a department or organization in charge of environmental work.	0.704		0.507
Our enterprise has a special budget for green production.	0.667		0.546
Our enterprise has a leadership that is committed to green production.	0.665		0.498
The sufficient capital level of our enterprise can support green production.	0.659		0.492
Our enterprise regularly trains employees in green production-related skills.	0.654		0.568
Our enterprise has sufficient talent reserves related to green production.	0.581		0.585
Our enterprise has production equipment that fully meets the needs of green production.	0.559		0.488
Our enterprise can easily design green ecological products.		0.795	0.681
Our corporate products have the ability to register the green logo.		0.790	0.654
Our enterprise has the ability to market green products.		0.771	0.642
In the field of green production, our enterprise has stocked related advanced technologies.		0.594	0.586
Eigenvalue	6.257	1.221	
Cumulative % of explanatory variance	48.130	57.522	

**Table 5:** Test results of the internal factors (IFs) sub-scale (*N* = 853).

Factor	Item	Corrected Item and Total Correlation	Number of Items	Cronbach’s α
IFs sub-scale			13	0. 908
Corporate tangible resources			9	0.888
	Our enterprise is an organization with a high awareness and mission of green production.	0.645		
	Our enterprise has a leadership that values green production.	0.660		
	Our enterprise has a department or organization in charge of environmental work.	0.548		
	Our enterprise has a special budget for green production.	0.658		
	Our enterprise has a leadership that is committed to green production.	0.601		
	The sufficient capital level of our enterprise can support green production.	0.597		
	Our enterprise regularly trains employees in green production-related skills.	0.685		
	Our enterprise has sufficient talent reserves related to green production.	0.704		
	Our enterprise has production equipment that fully meets the needs of green production.	0.633		
Corporate intangible resources			4	0.808
	Our enterprise can easily design green ecological products.	0.582		
	Our corporate products have the ability to register the green logo.	0.599		
	Our enterprise has the ability to market green products.	0.555		
	In the field of green production, our enterprise has stocked related advanced technologies.	0.686		

**Table 6:** Components and factor loadings of the external factors (EFs) sub-scale (*N* = 853).

Item	Component			Communality
	Market Environment	Public Supervision	Policy and Institutional Environment	
The enforcement of green production-related regulations in the market is strict.	0.700			0.586
The implementation of green production-related systems in the market is strict.	0.677			0.556
Consumers trust green products.	0.651			0.540
Green production helps to enhance corporate image and brand value.	0.632			0.507
Consumers tend to buy green products.	0.595			0.463
In the market, enterprises are heavily regulated.	0.593			0.440
In the market, the green production of enterprises has been actively supported.	0.550			0.443
In the market, enterprises’ participation in the construction of ecological industrial parks has been actively supported.	0.543			0.395
The customer (enterprise) has high requirements for the environment of our enterprise.	0.496			0.456
Investors place high demands on the environmental protection of our enterprise.	0.495			0.405
In the market, green production-related regulations and systems are highly practical.	0.487			0.465
Community residents are required to participate in the environmental impact approval process of surrounding enterprises.		0.712		0.578
The public and the community will make petition letters or complaints about environmental violations of surrounding enterprises.		0.701		0.525
Residents of the community require surrounding enterprises to build public environmental protection infrastructure.		0.687		0.533
Social environmental organizations are very concerned about corporate environmental violations.		0.684		0.547
News media will report on corporate environmental violations.		0.620		0.461
Peers are very concerned about enterprise green production capabilities.		0.540		0.367
Consumers pay great attention to the environmental violations of enterprises.		0.515		0.471
Green products have passed strict certification.		0.466		0.412
The government has developed preferential land policies for enterprises adopting clean technologies.			0.759	0.659
The government has developed investment and financing policies for enterprises adopting clean technologies.			0.741	0.656
The government has developed fiscal and tax incentives for enterprises adopting clean technologies.			0.710	0.599
The government actively implements preferential policies for cleaner production of enterprises.			0.700	0.581
Eigenvalue	8.389	1.707	1.548	
Cumulative % of explanatory variance	36.472	43.895	50.625	

**Table 7:** Test results of the EFs sub-scale (*N* = 853).

Factor	Item	Corrected Item and Total Correlation	Number of Items	Cronbach’s α
EFs sub-scale			23	0. 920
Market environment			11	0.869
	The enforcement of green production-related regulations in the market is strict.	0.533		
	The implementation of green production-related systems in the market is strict.	0.506		
	Consumers trust green products.	0.557		
	Green production helps to enhance corporate image and brand value.	0.559		
	Consumers tend to buy green products.	0.525		
	In the market, enterprises are heavily regulated.	0.534		
	In the market, the green production of enterprises has been actively supported.	0.560		
	In the market, enterprises’ participation in the construction of ecological industrial parks has been actively supported.	0.534		
	The customer (enterprise) has high requirements for the environment of our enterprise.	0.583		
	Investors place high demands on the environmental protection of our enterprise.	0.566		
	In the market, green production-related regulations and systems are highly practical.	0.587		
Public supervision			8	0.845
	Community residents are required to participate in the environmental impact approval process of surrounding enterprises.	0.542		
	The public and the community will make petition letters or complaints about environmental violations of surrounding enterprises.	0.523		
	Residents of the community require surrounding enterprises to build public environmental protection infrastructure.	0.570		
	Social environmental organizations are very concerned about corporate environmental violations.	0.593		
	News media will report on corporate environmental violations.	0.546		
	Peers are very concerned about the enterprise’s green production capabilities.	0.495		
	Consumers pay great attention to the environmental violations of enterprises.	0.615		
	Green products have passed strict certification.	0.576		
Policy and institutional environment			4	0.827
	The government has developed preferential land policies for enterprises adopting clean technologies.	0.544		
	The government has developed investment and financing policies for enterprises adopting clean technologies.	0.588		
	The government has developed fiscal and tax incentives for enterprises adopting clean technologies.	0.559		
	The government actively implements preferential policies for cleaner production of enterprises.	0.548		

**Table 8:** Component and factor loadings of the GDB-IE sub-scale (*N* = 853).

Item	Component		Communality
	Clean Production Behavior	Green Supply Chain Management Practices	
The production process of our enterprise strictly adheres to the requirements of cleaner production.	0.753		0.609
Our enterprise is selecting and improving pro-environmental processes or equipment.	0.745		0.583
Our enterprise purchases environmentally friendly processes and equipment.	0.735		0.570
Our enterprise considers the need for cleaner production when designing products.	0.724		0.576
Our enterprise actively builds a cleaner production brand.	0.642		0.499
Our enterprise has promoted the image of cleaner production.	0.610		0.467
Our enterprise cascades use energy between enterprises.	0.573		0.497
Our enterprise recycles water between enterprises.	0.534		0.440
Our enterprise is actively looking for partners to jointly achieve the goals of energy conservation and emission reduction.	0.529		0.465
Our enterprise actively recycles and disposes of waste products.	0.409		0.266
Our enterprise conducts environmental and energy audits on the internal management of suppliers.		0.812	0.672
Our enterprise requires suppliers to provide design specifications for the environmentally friendly requirements of the products they purchase.		0.770	0.634
Our enterprise evaluates suppliers’ environmentally friendly practices.		0.761	0.651
In the supply chain, our enterprise is very concerned about the green technologies of other enterprises.		0.613	0.599
Our enterprise purchases new energy-saving and low-carbon materials and new energy.		0.524	0.505
In the supply chain, our enterprise share energy-saving and emission-reduction technologies among enterprises actively.	s	0.518	0.490
Our enterprise chooses suppliers that have passed third-party environmental management system certification (e.g., ISO 14001).		0.509	0.312
In the supply chain, our enterprise communicates information about byproducts between enterprises actively.		0.500	0.447
Eigenvalue	7.996	1.287	
Cumulative % of explanatory variance	44.424	51.575	

**Table 9:** Reliability test results of the GDB-IE sub-scale (*N* = 853).

Factor	Item	Corrected Item and Total Correlation	Number of Items	Cronbach’s α
GDB-IE sub-scale			18	0.925
Clean production behavior			10	0.880
	The production process of our enterprise strictly adheres to the requirements of cleaner production.	0.648		
	Our enterprise is selecting and improving pro-environmental processes or equipment.	0.614		
	Our enterprise purchases environmentally friendly processes and equipment.	0.611		
	Our enterprise considers the need for cleaner production when designing products.	0.638		
	Our enterprise actively builds a cleaner production brand.	0.623		
	Our enterprise has promoted the image of cleaner production.	0.605		
	Our enterprise cascades use energy between enterprises.	0.651		
	Our enterprise recycles water between enterprises.	0.610		
	Our enterprise is actively looking for partners to jointly achieve the goals of energy conservation and emission reduction.	0.630		
	Our enterprise actively recycles and disposes of waste products.	0.465		
Green supply chain management practices			8	0.866
	Our enterprise conducts environmental and energy audits on the internal management of suppliers.	0.575		
	Our enterprise requires suppliers to provide design specifications for the environmentally friendly requirements of the products they purchase.	0.615		
	Our enterprise evaluates suppliers’ environmentally friendly practices.	0.659		
	In the supply chain, our enterprise is very concerned about the green technologies of other enterprises.	0.715		
	Our enterprise purchases new energy-saving and low-carbon materials and new energy.	0.659		
	In the supply chain, our enterprise actively shares energy-saving and emission-reduction technologies among enterprises.	0.646		
	Our company chooses suppliers that have passed third-party environmental management system certification (e.g., ISO 14001).	0.462		
	In the supply chain, our enterprise actively communicates information about byproducts between enterprises.	0.613		

**Table 10:** Component and factor loadings of the GDP-IE sub-scale (*N* = 853).

Item	Component			Communality
	Corporate Social Performance	Corporate Financial Performance	Corporate Environmental Performance	
Customers increasingly trust our products.	0.776			0.681
Our enterprise’s image and brand value have been enhanced.	0.755			0.64
Our enterprise has improved product quality.	0.638			0.532
Stakeholders have a high opinion of our enterprise.	0.636			0.563
Our enterprise has improved relationships with the people in our communities.	0.607			0.512
Our enterprise reduces the possibility of environmental accidents.	0.580			0.480
Our enterprise has reduced material costs.		0.824		0.716
Our enterprise has reduced operating costs.		0.799		0.703
Our enterprise has reduced energy costs.		0.746		0.647
Our enterprise has reduced the cost of environmental governance (e.g., emissions, penalties).		0.597		0.450
Our enterprise has improved long-term financial performance.		0.559		0.539
Our enterprise has reduced procurement costs through material recycling.		0.543		0.463
Wastewater emissions from our enterprise have decreased.			0.810	0.752
The emissions of exhaust gas produced by our enterprise have decreased.			0.779	0.719
The amount of solid waste produced by our enterprise has decreased.			0.702	0.652
Our enterprise has reduced the consumption of dangerous, toxic, and harmful substances.			0.547	0.537
Eigenvalue	7.007	1.614	0.964	
Cumulative % of explanatory variance	43.794	53.882	59.908	

**Table 11:** Test results of the GDP-IE sub-scale (*N* = 853).

Factor	Item	Corrected Item and Total Correlation	Number of Items	Cronbach’s α
GDP-IE sub-scale			16	0. 909
Corporate social performance			6	0.845
	Customers increasingly trust our products.	0.639		
	Our enterprise’s image and brand value have been enhanced.	0.618		
	Our enterprise has improved product quality.	0.610		
	Stakeholders have a high opinion of our enterprise.	0.648		
	Our enterprise has improved relationships with the people in our communities.	0.589		
	Our enterprise reduces the possibility of environmental accidents.	0.551		
Corporate financial performance				
	Our enterprise has reduced material costs.	0.583	6	0.833
	Our enterprise has reduced operating costs.	0.590		
	Our enterprise has reduced energy costs.	0.626		
	Our enterprise has reduced the cost of environmental governance (e.g., emissions, penalties).	0.445		
	Our enterprise has improved long-term financial performance.	0.637		
	Our enterprise has reduced procurement costs through material recycling.	0.593		
Corporate environmental performance			4	0.834
	Wastewater emissions from our enterprise have decreased.	0.611		
	The emissions of exhaust gas produced by our enterprise have decreased.	0.617		
	The amount of solid waste produced by our enterprise has decreased.	0.632		
	Our enterprise has reduced the consumption of dangerous, toxic, and harmful substances.	0.596		

## Appendix A

**Table A1:** Questionnaire items on the green development behavior and performance of industrial enterprises (GDBP-IE).

Sub-Scale	Dimensions	Codes	Items
Internal Factors (IFs)	Corporate tangible resources (CTR)	IFs1	Our enterprise is an organization with a high awareness and mission of green production.
		IFs2	Our enterprise has a leadership that values green production.
		IFs3	Our enterprise has a department or organization in charge of environmental work.
		IFs4	Our enterprise has a special budget for green production.
		IFs5	Our enterprise has a leadership that is committed to green production.
		IFs6	The sufficient capital level of our enterprise can support green production.
		IFs7	Our enterprise regularly trains employees in green production-related skills.
		IFs8	Our enterprise has sufficient talent reserves related to green production.
		IFs9	Our enterprise has production equipment that fully meets the needs of green production.
	Corporate intangible resources (CIR)	IFs10	Our enterprise can easily design green ecological products.
		IFs11	Our corporate products have the ability to register the green logo.
		IFs12	Our enterprise has the ability to market green products.
		IFs13	In the field of green production, our enterprise has stocked related advanced technologies.
External Factors (EFs)	Market environment (ME)	EFs1	The enforcement of green production-related regulations in the market is strict.
		EFs2	The implementation of green production-related systems in the market is strict.
		EFs3	Consumers trust green products.
		EFs4	Green production helps to enhance corporate image and brand value.
		EFs5	Consumers tend to buy green products.
		EFs6	In the market, enterprises are heavily regulated.
		EFs7	In the market, the green production of enterprises has been actively supported.
		EFs8	In the market, enterprises’ participation in the construction of ecological industrial parks has been actively supported.
		EFs9	The customer (enterprise) has high requirements for the environment of our enterprise.
		EFs10	Investors place high demands on the environmental protection of our enterprise.
		EFs11	In the market, green production-related regulations and systems are highly practical.
	Public supervision (PS)	EFs12	Community residents are required to participate in the environmental impact approval process of surrounding enterprises.
		EFs13	The public and the community will make petition letters or complaints about environmental violations of surrounding enterprises.
		EFs14	Residents of the community require surrounding enterprises to build public environmental protection infrastructure.
		EFs15	Social environmental organizations are very concerned about corporate environmental violations.
		EFs16	News media will report on corporate environmental violations.
		EFs17	Peers are very concerned about the enterprise’s green production capabilities.
		EFs18	Consumers pay great attention to the environmental violations of enterprises.
		EFs19	Green products have passed strict certification.
	Policy and institutional environment (PIE)	EFs20	The government has developed preferential land policies for enterprises adopting clean technologies.
		EFs21	The government has developed investment and financing policies for enterprises adopting clean technologies.
		EFs22	The government has developed fiscal and tax incentives for enterprises adopting clean technologies.
		EFs23	The government actively implements preferential policies for cleaner production of enterprises.
Green Development Behavior of Industrial Enterprises (GDB-IE)	Clean production behavior (CPB)	GDB-IE1	The production process of our enterprise strictly adheres to the requirements of cleaner production.
		GDB-IE2	Our enterprise is selecting and improving pro-environmental processes or equipment.
		GDB-IE3	Our enterprise purchases environmentally friendly processes and equipment.
		GDB-IE4	Our enterprise considers the need for cleaner production when designing products.
		GDB-IE5	Our enterprise actively builds a cleaner production brand.
		GDB-IE6	Our enterprise has promoted the image of cleaner production.
		GDB-IE7	Our enterprise cascades use energy between enterprises.
		GDB-IE8	Our enterprise recycles water between enterprises.
		GDB-IE9	Our enterprise is actively looking for partners to jointly achieve the goals of energy conservation and emission reduction.
		GDB-IE10	Our enterprise actively recycles and disposes of waste products.
	Green supply chain management practices (GSCMP)	GDB-IE11	Our enterprise conducts environmental and energy audits on the internal management of suppliers.
		GDB-IE12	Our enterprise requires suppliers to provide design specifications for the environmentally friendly requirements of the products they purchase.
		GDB-IE13	Our enterprise evaluates suppliers’ environmentally friendly practices.
		GDB-IE14	In the supply chain, our enterprise is very concerned about the green technologies of other enterprises.
		GDB-IE15	Our enterprise purchases new energy-saving and low-carbon materials and new energy.
		GDB-IE16	In the supply chain, our enterprise actively shares energy-saving and emission-reduction technologies among enterprises.
		GDB-IE17	Our company chooses suppliers that have passed third-party environmental management system certification (e.g., ISO 14001).
		GDB-IE18	In the supply chain, our enterprise actively communicates information about byproducts between enterprises.
Green Development Performance of Industrial Enterprises (GDP-IE)	Corporate social performance (CSP)	GDP-IE1	Customers increasingly trust our products.
		GDP-IE2	Our enterprise’s image and brand value have been enhanced.
		GDP-IE3	Our enterprise has improved product quality.
		GDP-IE4	Stakeholders have a high opinion of our enterprise.
		GDP-IE5	Our enterprise has improved relationships with the people in our communities.
		GDP-IE6	Our enterprise reduces the possibility of environmental accidents.
	Corporate financial performance (CFP)	GDP-IE7	Our enterprise has reduced material costs.
		GDP-IE8	Our enterprise has reduced operating costs.
		GDP-IE9	Our enterprise has reduced energy costs.
		GDP-IE10	Our enterprise has reduced the cost of environmental governance (e.g., emissions, penalties).
		GDP-IE11	Our enterprise has improved long-term financial performance.
		GDP-IE12	Our enterprise has reduced procurement costs through material recycling.
	Corporate environmental performance (CEP)	GDP-IE13	Wastewater emissions from our enterprise have decreased.
		GDP-IE14	The emissions of exhaust gas produced by our enterprise have decreased.
		GDP-IE15	The amount of solid waste produced by our enterprise has decreased.
		GDP-IE16	Our enterprise has reduced the consumption of dangerous, toxic, and harmful substances.

## References

[1] Brundtland G.H., Khalid M., Agnelli S., Al-Athel S., Chidzero B. (1987). Our Common Future.

[2] United Nations Development Programme Sustainable Development Goals. https://www.undp.org/content/undp/en/home/sustainable-development-goals.html.

[3] Li X., Du J., Long H. (2019). Theoretical framework and formation mechanism of the green development system model in China. Environ. Dev..

[4] Koutsoyiannis D. (2011). Scale of water resources development and sustainability: Small is beautiful, large is great. Hydrol. Sci. J..

[5] Sargentis G., Ioannidis R., Karakatsanis G., Sigourou S., Lagaros N.D., Koutsoyiannis D. (2019). The development of the Athens water supply system and inferences for optimizing the scale of water infrastructures. Sustainability.

[6] National Bureau of Statistics of China National Data. http://data.stats.gov.cn/easyquery.htm?cn=C01.

[7] Islam M., Managi S. (2019). Green growth and pro-environmental behavior: Sustainable resource management using natural capital accounting in India. Resour. Conserv. Recycl..

[8] Yusof N.A., Abidin N.Z., Zailani S.H.M., Govindan K., Iranmanesh M. (2016). Linking the environmental practice of construction firms and the environmental behaviour of practitioners in construction projects. J. Clean. Prod..

[9] National Bureau of Statistics of China China Statistical Yearbook 2019. http://www.stats.gov.cn/tjsj/ndsj/2019/indexch.htm.

[10] Liu H., Long H., Li X. (2020). Identification of critical factors in construction and demolition waste recycling by the grey-DEMATEL approach: A Chinese perspective. Environ. Sci. Pollut. Res..

[11] Li X., Du J., Long H. (2018). A Comparative Study of Chinese and Foreign Green Development from the Perspective of Mapping Knowledge Domains. Sustainability.

[12] Yuan Q., Yang D., Yang F., Luken R., Saieed A., Wang K. (2019). Green industry development in China: An index based assessment from perspectives of both current performance and historical effort. J. Clean. Prod..

[13] Jiahuey Y., Liu Y., Yu Y. (2019). Measuring green growth performance of China’s chemical industry. Resour. Conserv. Recycl..

[14] Feng C., Wang M. (2019). Journey for green development transformation of China’s metal industry: A spatial econometric analysis. J. Clean. Prod..

[15] Wang W., Yu B., Yan X., Yao X., Liu Y. (2017). Estimation of innovation’s green performance: A range-adjusted measure approach to assess the unified efficiency of China’s manufacturing industry. J. Clean. Prod..

[16] Chen Y.S., Lai S.B., Wen C.T. (2006). The influence of green innovation performance on corporate advantage in Taiwan. J. Bus. Ethics.

[17] Obeidat S.M., Al Bakri A.A., Elbanna S. (2018). Leveraging “Green” Human Resource Practices to Enable Environmental and Organizational Performance: Evidence from the Qatari Oil and Gas Industry. J. Bus. Ethics.

[18] Diabat A., Khodaverdi R., Olfat L. (2013). An exploration of green supply chain practices and performances in an automotive industry. Int. J. Adv. Manuf. Technol..

[19] Namagembe S., Ryan S., Sridharan R. (2019). Green supply chain practice adoption and firm performance: Manufacturing SMEs in Uganda. Manag. Environ. Qual..

[20] Chang T.-W., Chen F.-F., Luan H.-D., Chen Y.-S. (2019). Effect of Green Organizational Identity, Green Shared Vision, and Organizational Citizenship Behavior for the Environment on Green Product Development Performance. Sustainability.

[21] Steyrer J., Schiffinger M., Lang R. (2008). Organizational commitment—A missing link between leadership behavior and organizational performance?. Scand. J. Manag..

[22] Chiang C.-F., Hsieh T.-S. (2012). The impacts of perceived organizational support and psychological empowerment on job performance: The mediating effects of organizational citizenship behavior. Int. J. Hosp. Manag..

[23] Li X., Du J., Long H. (2019). Green Development Behavior and Performance of Industrial Enterprises Based on Grounded Theory Study: Evidence from China. Sustainability.

[24] Armento M.E., Hopko D.R. (2007). The environmental reward observation scale (EROS): Development, validity, and reliability. Behav. Ther..

[25] Robin M., Matheau-Police A., Couty C. (2007). Development of a scale of perceived environmental annoyances in urban settings. J. Environ. Psychol..

[26] Pluess M., Assary E., Lionetti F., Lester K.J., Krapohl E., Aron E.N., Aron A. (2018). Environmental sensitivity in children: Development of the Highly Sensitive Child Scale and identification of sensitivity groups. Dev. Psychol..

[27] Markle G.L. (2013). Pro-environmental behavior: Does it matter how it’s measured? Development and validation of the pro-environmental behavior scale (PEBS). Hum. Ecol..

[28] Homburg A., Stolberg A., Wagner U. (2007). Coping with global environmental problems: Development and first validation of scales. Environ. Behav..

[29] Powell R.B., Stern M.J., Krohn B.D., Ardoin N. (2011). Development and validation of scales to measure environmental responsibility, character development, and attitudes toward school. Environ. Educ. Res..

[30] Alisat S., Riemer M. (2015). The environmental action scale: Development and psychometric evaluation. J. Environ. Psychol..

[31] Ewert A., Galloway G. (2009). Socially desirable responding in an environmental context: Development of a domain specific scale. Environ. Educ. Res..

[32] Wernerfelt B. (1984). A Resource-Based View of the Firm. Strateg. Manag. J..

[33] Newbert S.L. (2007). Emprical Research on the Resource-based View of the Firm: An Assessment and Suggestions for Future Research. Strateg. Manag. J..

[34] Pohjola M. (2002). The new economy in growth and development. Oxf. Rev. Econ. Policy.

[35] Carmeli A., Tishler A. (2004). The Relationships between Intangible Organizational Elements and Organizational Performance. Strateg. Manag. J..

[36] Zhao Y., Fan B. (2018). Exploring open government data capacity of government agency: Based on the resource-based theory. Gov. Inf. Q..

[37] Hitt M.A., Xu K., Carnes C.M. (2016). Resource based theory in operations management research. J. Oper. Manag..

[38] Yang C.L. (2007). Model Establishment and Analysis of Enterprise Value Creation System. Stat. Decis..

[39] Orth R., Scheumann R., Galeitzke M., Wolf K., Kohl H., Finkbeiner M. (2015). Sustainable Corporate Development Measured by Intangible and Tangible Resources as Well as Targeted by Safeguard Subjects. Procedia CIRP.

[40] Newbert S.L. (2008). Value, Rareness, Competitive Advantage, and Performance: A Conceptual-level Empirical Investigation of the Resource-based View of the Firm. Strateg. Manag. J..

[41] Muller A., Kolk A. (2010). Extrinsic and Intrinsic Drivers of Corporate Social Performance: Evidence from Foreign and Domestic Firm in Mexico. J. Manag. Stud..

[42] Abeysekera I. (2019). How Best to Communicate Intangible Resources on Websites to Inform Corporate-Growth Reputation of Small Entrepreneurial Businesses. J. Small Bus. Manag..

[43] Surroca J., Tribó J.A., Waddock S. (2010). Corporate responsibility and financial performance: The role of intangible resources. Strateg. Manag. J..

[44] Helfat C.E., Peteraf M.A. (2003). The Dynamic Resource-based View: Capability Lifecycles. Strateg. Manag. J..

[45] Dong X., Wang T., Zhao C. (2016). Utility and Matching Measure Model of Firm Resources. Manag. Rev..

[46] North D.C. (1991). Institutions. J. Econ. Perspect..

[47] Li S., Zhang Y. (2009). Institutional Environment, Transaction Rule and Efficiency of Corporate Control Transfer. Econ. Res. J..

[48] Bain J.S. (1956). Barriers to New Competition.

[49] Bain J.S. (1959). Industrial Organization.

[50] Stigler G. (1968). The Organization of Industry.

[51] Jaiswal D., Kant R. (2018). Green purchasing behaviour: A conceptual framework and empirical investigation of Indian consumers. J. Retail. Consum. Serv..

[52] Zhu H. (2018). Policy Support, Supervision Response and Green Entrepreneurship of Leading Agricultural Enterprises. J. Northwest A&F Univ. Soc. Sci. Ed..

[53] Wang J. (2008). Research on the Correlation among Environmental Information Disclosure, Industry Differences and Supervisory System. Account. Res..

[54] Wu D. (2016). Corporate Governance, Media Coverage and Coporate Social Responsibility. J. Zhongnan Univ. Econ. Law.

[55] Porter M.E., Kramer M.R. (2006). Strategy and Society: The Link Between Competitive Advantage and Corporate Social Responsibility. Harv. Bus. Rev..

[56] Dyck A., Zingales L. (2004). Private Benefits of Control: An International Comparison. J. Financ..

[57] Dyck A., Luigi Z. (2008). The Corporate Governace Role of the Media: Evidence From Russia. J. Financ..

[58] Shiel C., Paço A.D., Alves H. (2020). Generativity, sustainable development and green consumer behaviour. J. Clean. Prod..

[59] Zhu N., Bu Y., Jin M., Mbroh N. (2020). Green Financial Behavior and Green Development Strategy of Chinese Power Companies in the Context of Carbon Tax. J. Clean. Prod..

[60] Silva D.A.L., Delai I., de Castro M.A.S., Ometto A.R. (2013). Quality tools applied to Cleaner Production programs: A first approach toward a new methodology. J. Clean. Prod..

[61] Vieira L.C., Amaral F.G. (2016). Barriers and strategies applying Cleaner Production: A systematic review. J. Clean. Prod..

[62] Jazairy A., von Haartman R. (2020). Analysing the institutional pressures on shippers and logistics service providers to implement green supply chain management practices. Int. J. Logist. Res. Appl..

[63] Cousins P.D., Lawson B., Petersen K.J., Fugate B. (2019). Investigating green supply chain management practices and performance. Int. J. Oper. Prod. Manag..

[64] Qin P., Zhao L., Wan J. (2014). The Impact of Cleaner Production Technology on Corporate Economic and Environmental Performance: An Empirical Study Based on the 2009 China Metal Products Industry Survey. Ecol. Econ..

[65] Mantovani A., Tarola O., Vergari C. (2017). End-of-pipe or cleaner production? How to go green in presence of income inequality and pro-environmental behavior. J. Clean. Prod..

[66] Crum M., Carter C.R., Liane Easton P. (2011). Sustainable supply chain management: Evolution and future directions. Int. J. Phys. Distrib. Logist. Manag..

[67] Ahi P., Searcy C. (2013). A comparative literature analysis of definitions for green and sustainable supply chain management. J. Clean. Prod..

[68] Rostamzadeh R., Govindan K., Esmaeili A., Sabaghi M. (2015). Application of fuzzy VIKOR for evaluation of green supply chain management practices. Ecol. Indic..

[69] Beamon B.M. (1999). Designing the green supply chain. Logist. Inf. Manag..

[70] Eltayeb T.K., Zailani S., Ramayah T. (2011). Green supply chain initiatives among certified companies in Malaysia and environmental sustainability: Investigating the outcomes. Resour. Conserv. Recycl..

[71] Rao P., Holt D. (2005). Do green supply chains lead to competitiveness and economic performance?. Int. J. Oper. Prod. Manag..

[72] Elkington J. (1994). Towards the Sustainable Corporation: Win-Win-Win Business Strategies for Sustainable Development. Calif. Manag. Rev..

[73] Shou Y., Shao J., Lai K., Kang M., Park Y. (2019). The impact of sustainability and operations orientations on sustainable supply management and the triple bottom line. J. Clean. Prod..

[74] Zafrilla J.-E., Arce G., Cadarso M.-Á., Córcoles C., Gómez N., López L.-A., Monsalve F., Tobarra M.-Á. (2019). Triple bottom line analysis of the Spanish solar photovoltaic sector: A footprint assessment. Renew. Sustain. Energy Rev..

[75] Biswas I., Raj A., Srivastava S.K. (2018). Supply chain channel coordination with triple bottom line approach. Transp. Res. Part E Logist. Transp. Rev..

[76] Ahmed W., Sarkar B. (2019). Management of next-generation energy using a triple bottom line approach under a supply chain framework. Resour. Conserv. Recycl..

[77] Zhu Q., Sarkis J., Lai K. (2007). Green supply chain management: pressures, practices and performance within the Chinese automobile industry. J. Clean. Prod..

[78] Zhu Q. (2012). A Resource-based View on Realization Mechanism of Regulations for Promoting Green Purchasing Practices among Manufacturers. Manag. Rev..

[79] Zhu Q., Yang Q. (2013). An Empirical Study on Enterprises’ Environmental Behaviors and Their Influencing Factors through Eco-industrial Parks Development in China. Manag. Rev..

[80] Zhu Q., Geng Y. (2006). Statistics Analysis on Types of Chinese Manufacturers Based on Practice of Green Supply Chain management and Their Performance. Appl. Stat. Manag..

[81] Xie X.B., Wu Y., Feng Z.L., Hao Z.T. (2015). Investigation of Green Behavior of Resource-based Enterprise in China. China Popul. Resour. Environ..

[82] Zhou S. (2011). Research on the Influencing Factors of Corporate Environmental Behavior. Stat. Decis..

[83] Zhu Q. (2009). An Empirical Study on Barriers for Implementing Green Supply Chain Management in Manufacturers. China Popul. Resour. Environ..

[84] Zhu Q., Feng Y., Choi S.B. (2017). The role of customer relational governance in environmental and economic performance improvement through green supply chain management. J. Clean. Prod..

[85] Cui H., Shi J. (2013). The Relationship between Open Innovation, Government Support and the Performance of Leading Agricultural Enterprises. Issues Agric. Econ..

[86] Kaiser H.F. (1974). An index of factorial simplicity. Psychometrika.

[87] Li X., Du J., Long H. (2019). Dynamic analysis of international green behavior from the perspective of the mapping knowledge domain. Environ. Sci. Pollut. Res..

